# Some Diseases of the Male Genital System

**Published:** 1908-02-15

**Authors:** 


### SOME DISEASES OF THE MALE GENITAL SYSTEM.

### VIII.—TUBERCULOUS DISEASE OF THE TESTIS AND EPIDIDYMIS.

One talks of tuberculous disease of the testis as if it were common for that organ to be primarily attacked by this disease. As a matter of fact, the testis is nearly always involved secondarily to the epididymis; and it is from here that the mischief spreads to the body of the testis and to other parts of the genital apparatus, more or less frequently to the prostate, and less commonly, perhaps, to the bladder. Like most forms of surgical tuberculosis, it must be regarded as a hæmic infection, in this case by means of the large branch of the spermatic artery which is given off to supply the epididymis. Nor is it unreasonable to expect that tuberculosis should manifest itself here commonly, because the terminal vessels of this artery are small and tortuous, and it is believed that surgical tuberculosis is the result of bacilli becoming, so to speak, impacted in small arteries and retained there as an embolus. As soon as this occurs they are able to start their deadly campaign upon the adjacent tissues. It is true that the epididymis itself is attacked in some cases secondarily to disease in other parts of the urinary apparatus, such as the kidney or the bladder. The writer must not be understood to imply, therefore, that the epididymis is necessarily the first part of the genital apparatus to be infected. This is, of course, not the case. The point is that when the body of the testis is involved, it will be almost invariably found that the epididymis shows more advanced signs of the disease.

Attempts have been made to prove that infection is, at any rate in some cases, the result of coitus with a woman suffering from tuberculous disease of the uterus or its appendages. Without going so far as to say that this absolutely cannot happen, it may be safely asserted that it cannot be a common event, if for no other reason than that tuberculosis of the female genital organs is comparatively rare, and although tuberculosis is an infective granuloma, and thus allied to syphilis, the method of infection is essentially different in the two diseases. Acquired syphilis is, as is known, the result of inoculation through an abraded surface, and many surgeons have been unfortunate enough to contract the disease in this way during the course of an operation. But this rarely, if ever, happens in the case of tuberculosis. It is true that there is a condition known as the verraca necrogenica or post-mortem porter's wart, but even in this case its interest lies mainly in its extreme rarity. This mode of causation may therefore be disregarded.

As in all tuberculosis lesions, injury may play an important part in predisposing to the disease. It is to be supposed that the great majority of individuals existing under modern social conditions possess tubercle bacilli in their blood-stream, which are inert as long as the normal vitality of the patient is sustained. But let the resistance of any organ become even momentarily impaired, as it is, for instance, by an injury: and what is the result. The bacilli are able to exert their evil influence upon the damaged tissues, and the outcome is—disease. For disease is the expression of a want of equilibrium between the vitality of the tissues and the virulence of the micro-organism. And this is true, not only of tubercle but of every other infective process. There is a case recorded which can only be explained on this hypothesis of two medical students who were attending a course of instruction at a fever hospital. They had gone through nearly the whole course without being infected by any of the diseases with which they came in contact. But one day they went round the wards after having played an exhausting game of tennis, and both of them contracted scarlet fever. Lobar pneumonia is another case in point. We all of us possess in our pharynx, according to the bacteriologists, the diplococcus pneumoniæ, but the micro-organism is unable to harm us as long as our general health is unimpaired. But if we are exposed to draughts and "catch a chill," as the lay phrase has it, we run the risk of incurring the disease. It is not the chill which causes the symptoms, but it predisposes us to infection by an ever-present enemy. And there is another point of interest about the relation of antecedent injury to the subsequent tuberculosis. It is often slight, so slight, in fact, as to have been ignored at the time by the patient as a matter of no moment, and only recalled subsequently. How are we to explain this? Volkmann has suggested that severe injuries are less likely to determine an outbreak of tuberculosis, because the reaction which they cause is sufficient to overcome the activity of the tubercle bacillus. The conclusion is, therefore, that tuberculosis of the testis is nearly always secondary to disease of the epididymis, and that it is a hæmic infection, often the result of some slight antecedent injury. Less commonly it follows on similar disease of some other part of the genito-urinary tract, for instance, the kidney.

The pathology of the condition does not differ from that of tuberculosis elsewhere. The disease starts as an isolated nodule in the epididymis, generally in the head, this being the part where the spermatic artery breaks up into its branches. This nodule enlarges, or there may originally be several small nodules, which enlarge until their margins are in contact, when they coalesce to form one focus. Sooner or later the centre caseates, so that a tuberculous abscess is formed. Its contents consist of the broken-down connective tissue cells which formed the nodule. Caseation is supposed to occur as the result of two factors: (i) the virulence of the toxins secreted by the tubercle bacilli, and (ii.) the avascularity of the tissue. In the course of time the testis is involved by direct extension of the process, and an exactly similar state of affairs is found in that organ. One or more abscesses may form in the body of the testis, and these eventually make their way to the surface, involving the skin of the scrotum, and discharging their contents externally. The tunica vaginalis is generally infected as well, so that there may be a co-existent hydrocele, as in all diseases of the body of the testis, or the tunica may even contain pus.

